# Effects of Familiar Language Lyrics in Self-Selected Motivational Music on Sprint Performance and Psychophysiological Responses: An Exploratory Study

**DOI:** 10.3390/jfmk10040446

**Published:** 2025-11-18

**Authors:** Shigeki Kasai, Daisuke Ando

**Affiliations:** 1 Integrated Graduate School of Medicine, Engineering, and Agricultural Sciences, University of Yamanashi, Kofu 400-8510, Japan; g19dim02@yamanashi.ac.jp; 2 Graduate School Department of Interdisciplinary Research, University of Yamanashi, Kofu 400-8510, Japan

**Keywords:** affective responses, auditory stimulation, autonomic nervous system, ergogenic aid, linguistic processing, prefrontal hemodynamics

## Abstract

**Background**: Listening to fast-tempo or motivational music before exercise may improve maximal pedaling performance, but the role of lyric comprehension remains unclear. This study tested whether self-selected music with familiar language lyrics perceived as motivational improves sprint performance and psychophysiological responses. **Methods**: Fourteen healthy young men (age: 19.7 ± 1.7 years; height: 171.2 ± 3.1 cm; weight: 65.8 ± 7.1 kg; body mass index: 22.4 ± 2.1 kg/m^2^) who exercised regularly (≥3 sessions/week) participated in a within-subject crossover design, completing a 20 s maximal pedaling test across 3 randomized conditions: control (Con; no music), music with unfamiliar language lyrics (UM), and self-selected music with familiar language lyrics (FM). All participants completed all three experimental conditions in a randomized order. Data were analyzed using repeated-measures ANOVA and paired *t*-tests (α = 0.05). Relative peak and mean power outputs were recorded. Motivation, arousal, and pleasant emotion were assessed at rest, after listening to music, after warm-up, and after exercise. Prefrontal hemodynamics and autonomic nervous system activity were recorded continuously. **Results**: Relative peak power was higher in FM (8.42 ± 0.61 W/kg) than Con (8.23 ± 0.58 W/kg; *p* < 0.01; *d* = 1.05); mean power did not differ across conditions. FM showed higher motivation and arousal after music compared with Con and UM, and higher pleasant emotion throughout. No significant differences across conditions were observed in prefrontal hemodynamics or autonomic nervous system activity. **Conclusions**: Listening to self-selected music with familiar language lyrics perceived as motivational during subsequent maximal pedaling enhanced peak power and psychological responses. Lyric comprehension and language familiarity likely play an important role in the ergogenic effects of music before high-intensity exercise.

## 1. Introduction

Athletes use music not only as a stimulant to elicit arousal, but also as a regulator of emotion to manage psychological states before competition [1]. The effects of music listening on athletic performance have been extensively studied [2]. Previous studies have shown that music enhances performance in both aerobic and anaerobic exercise modalities, such as treadmill running, cycling, sprinting, and resistance training [3,4,5]. These effects have been reported in trained and untrained individuals, including recreational exercisers, collegiate athletes, and professionals, although the magnitude of improvement often varies depending on training status and task characteristics [3,4].

Listening to music before or during exercise enhances motivation [6], and has also been shown to increase arousal and pleasant emotion, contributing to improved performance [2,4]. However, the mechanisms by which music exerts these effects are not fully understood.

Physiologically, music serves as an auditory stimulus that activates auditory pathways and limbic structures such as the amygdala, hippocampus, and nucleus accumbens [7]. Furthermore, these regions are closely linked with dopaminergic and noradrenergic systems that regulate arousal, motivation, and emotional responses. Activation of these neural circuits promotes the release of dopamine and catecholamines, thus enhancing cardiovascular arousal and motor readiness via the autonomic nervous system [7,8,9].

Moreover, lyric comprehension may influence the psychological effects of music. When lyrics are presented in a familiar language, their meaning and emotional content can be more deeply conveyed, enhancing listeners’ emotional connection and empathy with the music. This may elicit stronger motivational and emotional responses than when the lyrics are incomprehensible. Such effects are supported by recent findings showing that lyric content and comprehension modulate emotional and motivational responses to music [10,11]. Therefore, examining how lyric comprehension affects exercise performance and psychological responses is important for understanding the mechanisms underlying music-induced performance enhancement.

In addition to these physiological processes, individual and cultural factors also influence how music affects exercise responses. For instance, Priest and Karageorghis (2008) demonstrated that music preference, familiarity, and cultural relevance shape listeners’ emotional and motivational experiences during exercise [12]. Therefore, lyric comprehension in a familiar language—one that is personally meaningful or culturally resonant to the listener—may enhance emotional engagement and motivation compared to unfamiliar language lyrics [12].

Therefore, it is plausible that listening to motivational music induces both psychological and physiological activation that may contribute to improved performance. Thus, listening to music is thought to influence physical performance by acting on psychological aspects.

In recent years, sprint interval training (SIT) has attracted attention as an effective training method that can yield significant cardiovascular and metabolic benefits despite requiring substantially shorter exercise durations than traditional endurance training [13]. However, during high-intensity exercise, a decrease in pleasure and an increase in displeasure have been reported, which may reduce motivation to continue exercising [14]. Therefore, maintaining pleasure and suppressing displeasure are critical challenges during the implementation of SIT. Music listening may provide an effective strategy to address these psychological challenges [2,3,14], and examining its effects on short-duration, high-intensity exercise is particularly important. Furthermore, incorporating music from the training phase may help enhance sprint performance and, consequently, maximize training adaptations.

Music listening activates the mesolimbic reward system and stimulates dopamine release, thereby activating the prefrontal cortex [8,9]. On the other hand, music listening is associated with increased sympathetic nervous system activity by enhancing positive emotions and altering heart rate and other physiological parameters [15]. Furthermore, listening to fast-tempo music and the emotional and physiological arousal induced by music listening increase plasma catecholamine concentrations [16,17]. These findings indicate that music listening induces physiological changes. These autonomic and neurochemical responses regulate cardiovascular, respiratory, and motor outputs to prepare the body for physical activity [18].

Previous studies have reported that listening to fast-tempo music may enhance positive emotions and arousal, thereby contributing to improved exercise performance. For instance, listening to fast-tempo music during passive rest between maximal pedaling bouts was shown to increase subsequent peak and mean power [19]. In other research, listening to motivational fast-tempo music has been associated with enhanced positive affect and increased power output in sprint tasks [20]. Likewise, preferred fast-tempo music has been linked to elevated motivation and improved performance in resistance exercise contexts [21]. A critical insight from Dobashi et al. (2021) is that the increase in arousal induced by fast-tempo music correlates with improvements in sprint performance [19]. Moreover, music listening, in general, enhances arousal, and fast-tempo music tends to evoke a greater positive affective state [22]. Taken together, these findings support the view that music listening can enhance exercise performance by increasing positive affect, arousal, and motivation.

Music that enhances motivation during exercise and sport typically possesses a fast tempo (generally above 120 beats per minute [bpm]), appealing melodies, emotionally charged or meaningful lyrics, associations with the sporting endeavor, and bright, uplifting, and harmonious structures [2,23]. These musical characteristics are considered to enhance motivation either individually or synergistically. Numerous previous studies have examined the effects of fast-tempo music. Among the elements constituting music, lyrics are not merely an additional component but play a decisive role in the impact of music on mood and emotion [24], and the content of lyrics has been shown to influence both emotions and cognition [25]. Furthermore, music may enhance motivation [26], and it has been reported that lyrics directly influence exercise performance [27]. On the other hand, listening to music preferred by the individual has been suggested to improve sprint and running performance [28,29,30], and preferred music inherently possesses characteristics that enhance motivation [31]. Therefore, when examining the effects of music listening on exercise performance, it is considered important to use music that increases motivation and is self-selected by the participants. Thus, the effect of lyrics is thought to be influenced by language comprehension; if the meaning or content of the lyrics cannot be understood, the effect of the lyrics may be reduced.

These previous studies suggest that music listening enhances athletic performance through psychological changes. Fast-tempo music and personally preferred music influence athletic performance by enhancing motivation, positive emotion, and arousal, while lyrics may play a significant role in mood and emotion. However, the effects of language comprehension of lyrics on sprint performance and physiological/psychological aspects, particularly the differences between familiar and unfamiliar languages on sprint performance, remain to be fully elucidated.

Therefore, this study aimed to examine the effects of self-selected music with familiar language lyrics perceived as motivational on sprint performance and psychophysiological responses during a 20 s maximal pedaling test under 3 randomized conditions.

## 2. Materials and Methods

This study employed a randomized, within-subject crossover design. Data collection was performed at the Human Performance Laboratory, University of Yamanashi (Kofu, Japan). All experimental procedures were approved by the Research Ethics Committee of the Faculty of Education, Graduate School Department of Interdisciplinary Research, University of Yamanashi (Approval No. R5-029) and were conducted in accordance with the principles of the Declaration of Helsinki. This study was not registered as a clinical trial, as it did not involve any medical intervention or patient enrollment, and was conducted solely with healthy volunteers.

### 2.1. Participants

Fourteen male university students with regular exercise habits participated in this study (mean ± SD: age, 19.7 ± 1.7 years; height, 171.2 ± 3.1 cm; weight, 65.8 ± 7.1 kg; body mass index, 22.4 ± 2.1 kg/m^2^). Participants were recruited through convenience sampling from the researchers’ personal and local networks within the university. Although no formal inclusion or exclusion criteria were established, participants were limited to healthy male university students with regular exercise habits and no self-reported history of cardiovascular, neurological, or musculoskeletal disorders. Health status and exercise eligibility were verbally confirmed by the researchers before participation. Exercise habits were confirmed through a researcher-administered questionnaire, indicating that all participants engaged in physical exercise at least 3 times per week. Height and body weight were measured using a stadiometer (Seca 217, Seca, Hamburg, Germany) and a digital scale (Seca 878, Seca, Hamburg, Germany). Body mass index was calculated as body mass (kg) divided by height squared (m^2^), providing a general indicator of body composition. Body composition was not assessed in this study.

A priori power analysis was conducted using G*Power (version 3.1.9.2) to determine an appropriate sample size for repeated-measures ANOVA. The analysis parameters were set as follows: effect size f = 0.40, α = 0.05, power (1 − β) = 0.80, 3 measurements, and correlation among repeated measures of 0.5. The analysis indicated that a sample size of 12 participants was required. However, to account for potential dropouts, 14 participants were recruited.

All participants received a detailed explanation of the study procedures and content in advance, and provided written informed consent. They completed all 3 experimental conditions (Con, UM, and FM) in a randomized order.

### 2.2. Experimental Method

In this study, each participant completed exercise testing under 3 conditions: control (Con; no music), listening to music with unfamiliar language lyrics (UM), and listening to self-selected music with familiar language lyrics perceived as motivational (FM).

**Figure 1:**
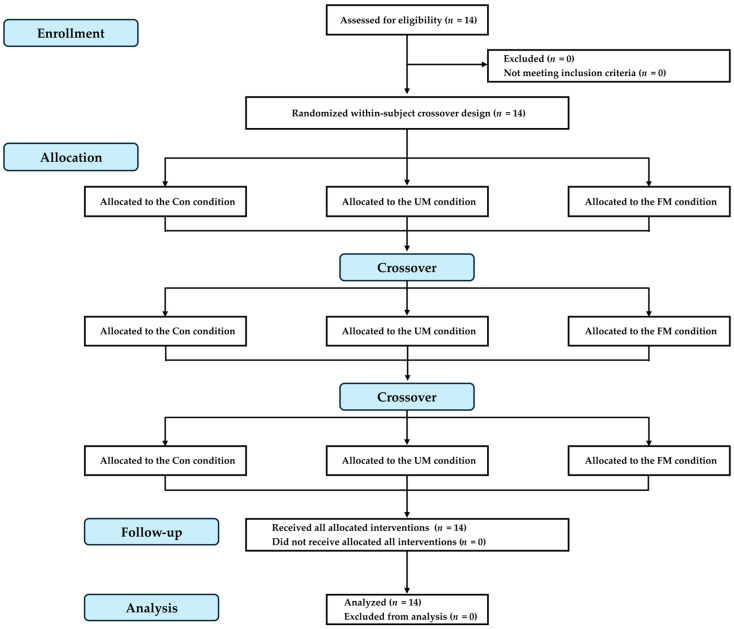
Flow diagram of the study. Participants completed 3 randomized conditions: control (Con; no music), listening to music with unfamiliar language lyrics (UM), and listening to self-selected music with familiar language lyrics perceived as motivational (FM). Each session was separated by at least 3 days to minimize potential carryover effects.

This study employed a crossover design where the same participants underwent exercise testing under the 3 different conditions. To counterbalance the conditions, the order of each condition was randomized. Testing was scheduled within the same time period for each condition within each participant. A wash-out period of at least 3 days was maintained between consecutive experimental trials, and the same minimum interval was established between the familiarization session and the first experimental trial. The flow diagram of the study is shown in Figure 1.

Warm-up and exercise testing were conducted using the POWER MAX V3 CONNECT (Konami Corp., Tokyo, Japan). Prior to the exercise test, participants rested in a seated position for 300 s. Following the rest period, they were exposed to their assigned condition for 180 s (Con, UM, or FM). After the conditioning period, they performed a 30 s low-intensity warm-up at 60 watts (with the bicycle load set to 1 kp and pedaling at 60 revolutions per minute). The exercise test consisted of a single 20 s maximal pedaling test. Exercise initiation followed a countdown starting from “5” displayed on the bicycle ergometer screen, synchronized with the start signal sound accompanying the ‘Start’ display.

**Figure 2:**
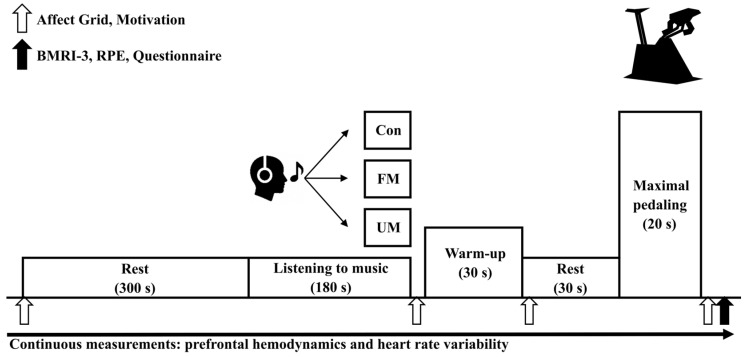
Experimental protocol and measurement time points. Participants completed 3 randomized conditions: control (Con; no music), listening to music with unfamiliar language lyrics (UM), and listening to self-selected music with familiar language lyrics perceived as motivational (FM). Mean tempo of the music with unfamiliar language lyrics (135 bpm; the value represents the actual measured tempo of the representative music) and the self-selected music with familiar language lyrics perceived as motivational (133.8 ± 2.8 bpm).

Bicycle load during the exercise test was calculated as “participant’s weight × 0.05” (unit: kp) and adjusted for each participant accordingly [20]. The handlebar height, grip position, and saddle height were kept identical across conditions within each participant, and were individually adjusted among participants according to their body size. The experimental protocol is shown in Figure 2.

All tests were conducted individually in a quiet, temperature-controlled laboratory at a mean temperature of 24.2 °C and a mean relative humidity of 65.8%. Illuminance in the laboratory, measured using a portable lux meter, ranged from 300 to 500 lx, corresponding to typical indoor lighting levels. An “Experiment in progress” sign was displayed outside to prevent entry or disturbance during testing, and background noise was checked to confirm a silent environment. External light was blocked with blackout curtains, and only ceiling lights were used for illumination. One participant and one investigator were present in the room during each session.

Participants were partially blinded to the study objectives; they were informed that the study examined the effects of different pre-exercise listening conditions on performance, but were unaware of the specific hypotheses or expected effects of each condition.

After arriving at the laboratory, participants were seated in a chair and remained there until the experiment began. The measuring instruments were then attached, and the experiment started immediately after instrumentation.

Each session included rest (300 s), listening to music (180 s after rest), warm-up (30 s), rest (30 s), and maximal pedaling (20 s). Immediately after the warm-up, participants were instructed to pedal “as hard and as fast as possible” during the 20 s maximal pedaling test, but received no verbal encouragement from the investigators to prevent external motivational bias.

Music was provided only after the initial rest period and before exercise, and was not played during the pedaling task. Psychological indices were assessed at the indicated time points: motivation, pleasant emotion, arousal, Brunel Music Rating Inventory-3 (BMRI-3), Rating of Perceived Exertion (RPE), and a questionnaire assessing music preference, lyric comprehension, language recognition, and lyric-related motivation. The lyric-related motivation item was rated on a 7-point scale, while other questionnaire items were answered dichotomously (Yes/No). Prefrontal hemodynamics and heart rate variability were continuously recorded throughout each session.

### 2.3. Music Selection and Listening

For the FM condition, participants listened to music they personally selected that they felt “increased motivation in a familiar language”. Familiar languages were defined as the participant’s native language (Japanese) and English, which is frequently studied in Japanese schools. As a practical approach that considered participants’ musical preferences, they were instructed to self-select “music in a familiar language” prior to the experiment. Specifically, participants were instructed to select 3 pieces of music that they felt would motivate them during maximal pedaling by listening to them before the exercise, and to rank the 3 pieces of music from No. 1 to No. 3. They were then asked to rank these 3 songs from 1st to 3rd place. From these 3 songs, the researcher selected one song with a tempo between 120 and 150 bpm for use in the experiment. If no song matching this tempo was available, the participant was asked to select an additional song they felt was motivating in a familiar language, and that song was used in the experiment. Consequently, the music listened to in the experiment differed among participants, with a mean tempo of 133.8 ± 2.8 bpm. Meanwhile, the UM condition used music in an unfamiliar language selected by the researchers. The unfamiliar language was Hindi, chosen because while it ranks high globally in terms of speaker population, exposure to it in daily life or school education is rare in Japan, making it accessible only through specialized educational institutions or courses. Hindi music was selected from songs ranked within the top 10 of Apple Inc.’s “Apple Music Top 100 Songs of 2023 India”. The researcher selected one song with a tempo close to the average tempo chosen by the participants (Riar Saab, Sharma A. Obsessed [Song]. Gully Gang/Mass Appeal. 2022., 135 bpm).

In the Con condition, no music was played after the seated rest period, whereas in the FM and UM conditions, music was delivered using a wireless Bluetooth speaker. The volume of the music listened to by the participants was standardized to approximately 60 dB, and the music listening time was set to 180 s.

### 2.4. Dietary and Lifestyle Instructions

Participants were instructed to maintain their usual lifestyle prior to the experiment. They were also instructed to keep their regular sleep and eating habits.

### 2.5. Familiarization Session

The familiarization session was conducted to adjust the pedals and saddle height of the POWER MAX V3 CONNECT used in the exercise test and to allow participants to become accustomed to the pedaling motion. Additionally, for each psychological measure, the familiarization session was also conducted to familiarize participants with the response methods, ensuring they could respond smoothly during the experiment.

### 2.6. Measurement Items

#### 2.6.1. Peak Power and Mean Power

As an exercise test, participants performed one 20 s maximal pedaling effort using the POWER MAX V3 CONNECT. The values measured by the device were recorded as peak power and mean power. Additionally, using each participant’s body weight data, relative peak power and relative mean power (W/kg) were calculated and used for analysis.

#### 2.6.2. Motivation

Motivation was evaluated using a questionnaire during seated rest, after music, after warm-up, and after the exercise test (Figure 2). Participants assessed their level of motivation for maximal pedaling using a Visual Analog Scale (VAS).

The VAS is a visual scale that allows participants to indicate their perceived state at the time of response. In this study, a 10 cm black line was used, with the right end representing “very high” and the left end “very low,” and participants were asked to mark the appropriate point. For data analysis, the distance from the left end was measured. During the familiarization session, participants were instructed on how to use the VAS and practiced marking sample responses to become accustomed to the task and the meaning of the scale anchors before the experimental trials.

Using ratings obtained during seated rest and after music, ΔMotivation = After music − Rest was computed (unitless). The VAS was adopted as a simple and validated measure that allows for sensitive assessment of transient motivational states in psychological and exercise-related research [5,26,27].

#### 2.6.3. Pleasant Emotion and Arousal

Pleasant emotion and arousal as represented by the Affect Grid were measured during seated rest, after music, after warm-up, and after the exercise test (Figure 2) [32]. The Affect Grid enables participants to visually and directly express their levels of pleasant emotion and arousal, allowing for rapid assessment. Previous studies using the Affect Grid have reported associations between arousal levels and sprint performance [19], suggesting that the Affect Grid is useful for evaluating pleasant emotion and arousal during exercise testing. The Affect Grid assesses affect along two dimensions: pleasure–displeasure and arousal–sleepiness. The horizontal-axis rating (pleasure–displeasure; 1–9, neutral = 5) was taken as the pleasant emotion score, and the vertical-axis rating (arousal–sleepiness; 1–9) as the arousal score, with higher values indicating greater levels on each dimension. Participants were instructed to select and mark the position on a 9 × 9 grid that best corresponded to their perceived levels of pleasant emotion and arousal.

Using ratings obtained during seated rest and after music, ΔPleasant Emotion = After music − Rest and ΔArousal = After music − Rest were computed (unitless). The Affect Grid was adopted because it is a simple, validated measure that enables rapid assessment of affective valence and arousal, making it suitable for repeated evaluations during experimental sessions.

#### 2.6.4. Motivational Components of Music and Lyrics

Using the Brunel Music Rating Inventory-3 (BMRI-3) [2,33], six musical factors—rhythm of the music, style of the music, melody of the music, tempo of the music, beat of the music, and sound of the instruments used—were scored on a 7-point scale ranging from 1 (“strongly disagree”) to 7 (“strongly agree”). The motivational components of the music listened to were assessed after the exercise test, and the total score for these six items was calculated (Figure 2). This measure was used to objectively assess the motivational components inherent in the music itself under each music condition. Additionally, the motivational component of the lyrics was assessed by asking participants to respond to the question “Did the lyrics of the music you listened to increase your motivation?” on a 7-point scale ranging from 1 (“strongly disagree”) to 7 (“strongly agree”) after the exercise test (Figure 2).

For the FM condition, questionnaires were administered after the exercise test to assess:

Q1. Did you like the music you listened to?

Q2. Did you understand the lyrics of the music?

For the UM condition, questionnaires were administered after the exercise test to assess:

Q1. Did you like the music you listened to?

Q2. Did you understand the lyrics of the music?

Q3. Did you recognize what language the lyrics were in?

#### 2.6.5. Perceived Exercise Intensity

Perceived exercise intensity was assessed using the Rating of Perceived Exertion (RPE) after the exercise test (Figure 2). Participants rated their perceived exertion using the Japanese version of the Borg Scale [34], which consists of 15 levels ranging from 6 to 20. In the original Borg Scale [35], a score of 6 corresponds to “no exertion at all”, while a score of 20 corresponds to “maximal exertion”. The Japanese version used in this study does not include these verbal labels but uses the same 6–20 numerical rating format as the original version.

#### 2.6.6. Prefrontal Hemodynamics

Prefrontal hemodynamics were measured using the Brite MK III to determine concentrations of oxygenated hemoglobin (HbO_2_, unit: mM·mm) and deoxygenated hemoglobin (HbR, unit: mM·mm) in the frontal region during seated rest and while listening to music.

To ensure consistency in anatomical positioning within the frontal region, probes were placed according to the international 10–20 system, using the frontal pole electrode positions Fp1 and Fp2 as reference points. The distance between the light-emitting and light-receiving probes was set to 3 cm to secure an appropriate penetration depth of near-infrared light. The region of interest (ROI) was defined as the medial prefrontal cortex (mPFC), including the frontopolar cortex (FPC) located at its most anterior part. Only the data corresponding to these channels were analyzed. Anatomical localization of each channel was performed using probabilistic registration [36,37].

Measurement data were extracted using OxySoft (Artinis Medical Systems B.V., Elst, The Netherlands), and analyzed in Python (version 3.13.1;Python Software Foundation, https://www.python.org/, accessed on 13 November 2025) using *the MNE-Python library* (version 1.10.1) [38] and *MNE-NIRS package* (version 0.7.1) [39] to ensure standardized preprocessing and analysis. After converting the light intensity data into optical density signals, a moving average filter (window width: 3.0 s) and a bandpass filter (0.01–0.5 Hz) were applied to remove high-frequency noise and physiological drift [40]. Furthermore, multi-resolution analysis using the discrete wavelet transform was performed to reduce physiological noise [41,42]. Subsequently, the Modified Beer–Lambert Law [43] was applied. Based on this, the optical pathlength factor was set to 6.0 for both wavelengths (760 nm and 850 nm) as a representative value for the adult forehead, and concentration changes in HbO_2_ and HbR were calculated [44].

Motion artifacts were detected using a threshold of five times the standard deviation of each channel signal. Continuous segments lasting for 3 s or longer were excluded from analysis, whereas minor artifacts shorter than 3 s were corrected by linear interpolation based on the average signal over the preceding and following 1 s intervals [45]. Previous studies have reported that cerebral blood flow dynamics stabilize approximately 5–10 s after stimulus onset [46,47]. Considering this stabilization period, the mean values of data recorded between 120 and 180 s from the start of measurement during seated rest and between 60 and 120 s during music listening were used to obtain stable signal segments. As baseline corrections, ΔHbO_2_ (After music − Rest) and ΔHbR (After music − Rest) were calculated. Although short-separation channels were recorded in all experiments, insufficient signal stability in some participants precluded their inclusion in the final analysis. All reported results are based on long-separation channel data.

#### 2.6.7. Autonomic Nervous System Activity

Heart rate variability (HRV) was continuously recorded using a wearable heart rate sensor (WHS-1, Union Tool, Tokyo, Japan) and analyzed using dedicated HRV analysis software (Acc Analyzer, GMS, Tokyo, Japan). Baseline HRV data were collected in a seated position to minimize postural effects on autonomic activity and to ensure participant comfort during the pre-exercise resting phase. This approach is consistent with established methodological recommendations and previous studies that have validated short-term HRV assessments under resting and pre-exercise conditions within exercise-related research contexts [48,49].

The low-frequency/high-frequency ratio (LF/HF, arbitrary units) was calculated as an index of autonomic nervous system (ANS) activity using frequency-domain analysis. Heart rate was continuously recorded throughout each experimental condition, from the resting period to the end of exercise, and HRV was subsequently analyzed based on these data. ΔLF/HF was computed as the difference between the values obtained during music listening and resting periods (ΔLF/HF = Music − Rest).

The measurement and analysis procedures followed the general recommendations of the Task Force of the European Society of Cardiology and the North American Society of Pacing and Electrophysiology [48]. The frequency-domain analysis approach used in this study is consistent with validated short-term HRV analytical methods reported in the previous literature [50]. The LF/HF ratio was selected as it reflects sympathovagal balance and is widely used as a practical indicator of autonomic modulation during psychophysiological responses to music and exercise [51,52].

### 2.7. Statistical Analyses

The normality of the data distribution was first verified using the Shapiro–Wilk test. Results are presented as mean ± standard error (SEM). For the main analyses, differences across conditions were examined using one-way repeated-measures ANOVA for peak power, mean power, ΔMotivation, ΔPleasant emotion, ΔArousal, ΔHbO_2_, ΔHbR, ΔLF/HF and RPE. A two-way repeated-measures ANOVA with condition (Con, UM, FM) and time (Rest, After Music, After Warm-up, After Exercise) as within-subject factors was used for motivation, pleasant emotion, and arousal. When a significant main effect or interaction was found, pairwise post hoc comparisons were performed using paired *t*-tests. For the BMRI-3 and the motivational component of the lyrics, differences across FM and UM conditions were analyzed using paired *t*-tests. All *p*-values were adjusted for multiple comparisons using the Benjamini–Hochberg method. Effect sizes were presented as partial *η*^2^ (*ηp*^2^) for ANOVA and Cohen’s d (*d*) for *t*-tests (small: 0.01, medium: 0.06, large: 0.14 for *ηp*^2^ [53]; small: 0.20, medium: 0.50, large: 0.80 for *d* [54]). Statistical significance was set at *p* < 0.05. All analyses were conducted using R (version 4.1.2; R Foundation for Statistical Computing, Vienna, Austria).

## 3. Results

The characteristics of the participants are summarized in Table 1. All participants were healthy young men who regularly engaged in physical exercise (≥3 sessions per week).

### 3.1. Peak Power

**Figure 3:**
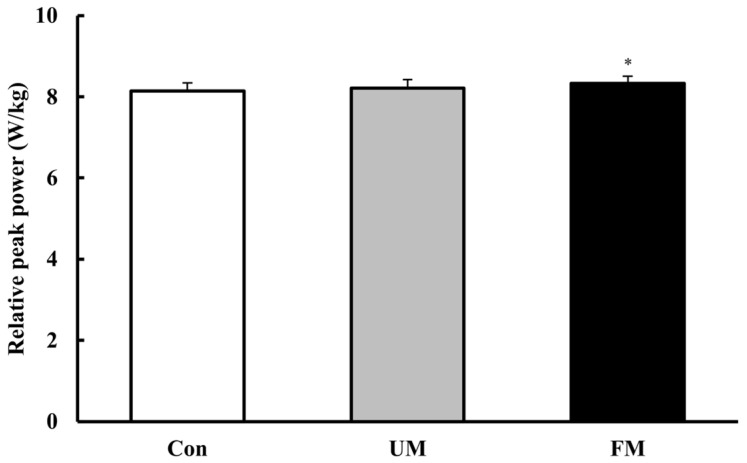
Relative peak power (W/kg) across the 3 music conditions. Values are presented as mean ± SEM (*n* = 14). A one-way repeated-measures ANOVA revealed a significant difference across conditions (*p* < 0.05). *: *p* < 0.01 vs. Con. Con: control; UM: unfamiliar language music; FM: familiar language music. Bar colors indicate stimulation level: white (Con), gray (UM), and black (FM).

Figure 3 shows relative peak power (W/kg) across the 3 music conditions. Analysis of variance revealed a significant difference across conditions (*F*(2, 26) = 4.93, *p* < 0.05, *ηp*^2^ = 0.28). Post hoc comparisons showed that the FM condition yielded significantly higher values than the Con condition (*M*_diff_ = 0.19, *p* < 0.01, *d* = 1.05). Although not statistically significant, the FM condition tended to be higher than the UM condition (*M*_diff_ = 0.12, *p* = 0.095, *d* = 0.54).

### 3.2. Mean Power

**Figure 4:**
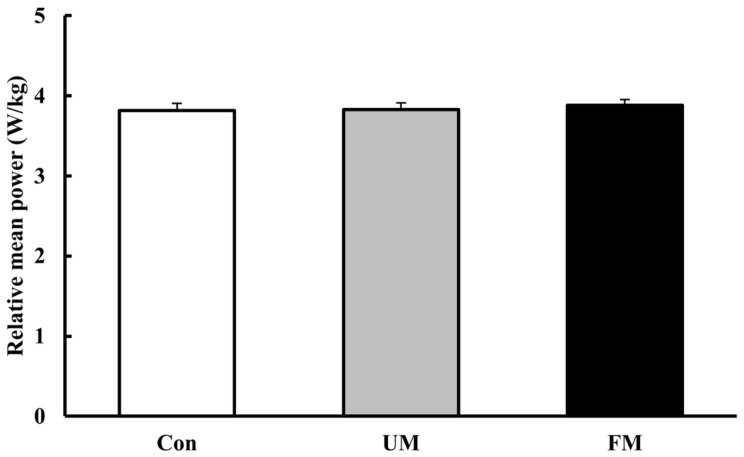
Relative mean power (W/kg) across the 3 music conditions. Values are presented as mean ± SEM (*n* = 14). A one-way repeated-measures ANOVA revealed no significant differences across conditions (*p* = 0.096). Con: control; UM: unfamiliar language music; FM: familiar language music. Bar colors indicate stimulation level: white (Con), gray (UM), and black (FM).

Figure 4 shows the relative mean power (W/kg) across the 3 music conditions. Analysis of variance revealed no significant differences across conditions (*F*(2, 26) = 2.57, *p* = 0.096, *ηp*^2^ = 0.17).

### 3.3. Motivation

**Figure 5:**
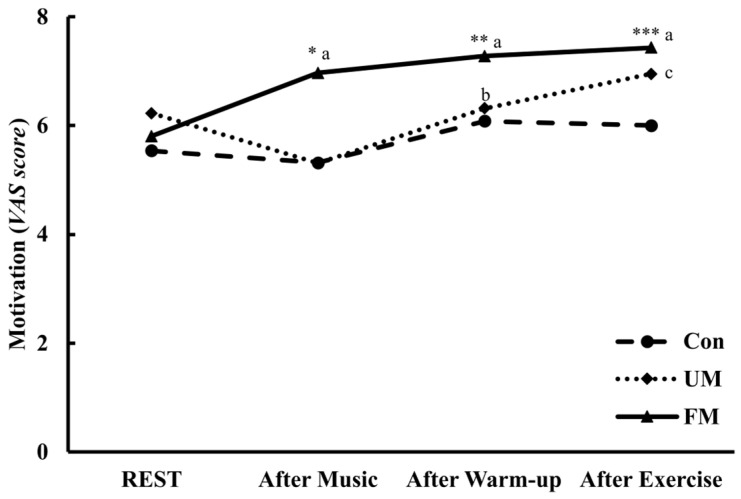
Changes in motivation (VAS, 0–100 mm) across the 3 music conditions and time points. Values are presented as mean (*n* = 14). Two-way repeated-measures ANOVA revealed a significant condition × time interaction (*p* < 0.001). *: *p* < 0.01 vs. Con and UM; **: *p* < 0.05 vs. Con and UM; ***: *p* < 0.05 vs. Con; a: *p* < 0.01 vs. Rest; b: *p* < 0.01 vs. After Music; c: *p* < 0.05 vs. After Music. Con: control; UM: unfamiliar language music; FM: familiar language music. Rest: at rest; After Music: after music listening; After Warm-up: after warm-up; After Exercise: after a 20 s maximal pedaling.

Figure 5 shows the temporal changes in motivation (VAS, 0–100 mm) across the 3 music conditions and time points. Analysis of variance revealed a significant interaction across music condition and time (*F*(6, 78) = 4.85, *p* < 0.001, *ηp*^2^ = 0.27).

The simple main effect of music condition was significant for After Music (*F*(2, 26) = 10.38, *p* < 0.01, *ηp*^2^ = 0.44), After Warm-up (*F*(2, 26) = 5.28, *p* < 0.05, *ηp*^2^ = 0.29), and After Exercise (*F*(2, 26) = 5.31, *p* < 0.05, *ηp*^2^ = 0.29). Post hoc comparisons revealed that in the After Music condition, the FM condition was significantly higher than both the Con (*M*_diff_ = 1.65, *p* < 0.01, *d* = 1.04) and UM conditions (*M*_diff_ = 1.66, *p* < 0.01, *d* = 1.03). In After Warm-up, the FM condition was significantly higher than the Con (*M*_diff_ = 1.19, *p* < 0.05, *d* = 0.67) and UM conditions (*M*_diff_ = 0.96, *p* < 0.05, *d* = 0.79), and in the After Exercise condition, the FM condition was significantly higher than the Con condition (*M*_diff_ = 1.43, *p* < 0.05, *d* = 0.77).

The simple main effect of time was significant in both the FM (*F*(3, 39) = 9.41, *p* < 0.001, *ηp*^2^ = 0.42) and UM conditions (*F*(3, 39) = 6.59, *p* < 0.01, *ηp*^2^ = 0.34). Post hoc multiple comparisons revealed that in the FM condition, After Music (*M*_diff_ = 1.16, *p* < 0.01, *d* = 1.00), After Warm-up (*M*_diff_ = 1.47, *p* < 0.01, *d* = 1.19), and After Exercise (*M*_diff_ = 1.63, *p* < 0.01, *d* = 0.97) were significantly higher than Rest. Similarly, in the UM condition, After Warm-up (*M*_diff_ = 1.01, *p* < 0.01, *d* = 1.10) and After Exercise (*M*_diff_ = 1.64, *p* < 0.05, *d* = 0.93) were significantly higher than After Music.

Analysis of variance revealed a significant difference across conditions for ΔMotivation (*F*(2, 26) = 10.28, *p* < 0.001, *ηp*^2^ = 0.44), and post hoc comparisons indicated that the FM condition was significantly higher than the Con (*M*_diff_ = 1.39, *p* < 0.01, *d* = 0.87) and UM conditions (*M*_diff_ = 2.08, *p* < 0.01, *d* = 1.03).

### 3.4. Pleasant Emotion

**Figure 6:**
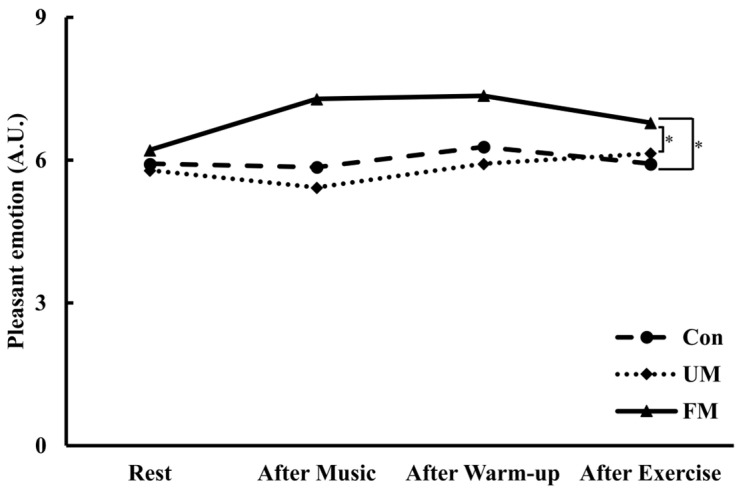
Changes in pleasant emotion (A.U.) across the 3 music conditions and time points. Values are presented as mean (*n* = 14). Two-way repeated-measures ANOVA revealed no significant condition × time interaction (*p* = 0.078), but a significant main effect of condition (*p* < 0.01). *: *p* < 0.01 vs. Con and UM. Con: control; UM: unfamiliar language music; FM: familiar language music. Rest: at rest; After Music: after music listening; After Warm-up: after warm-up; After Exercise: after a 20 s maximal pedaling.

Figure 6 shows the temporal changes in pleasant emotion (A.U.) across the 3 music conditions and time points. Although the interaction across condition and time did not reach statistical significance (*F*(6, 78) = 1.98, *p* = 0.078, *ηp*^2^ = 0.13), simple main effect analyses revealed significant differences across conditions at the After Music and After Warm-up time points. Post hoc comparisons indicated that at After Music, the FM condition was significantly higher than the Con (*M*_diff_ = 1.43, *p* < 0.01, *d* = 0.95) and UM conditions (*M*_diff_ = 1.86, *p* < 0.01, *d* = 0.87). Similarly, at After Warm-up, the FM condition was significantly higher than the Con (*M*_diff_ = 1.07, *p* < 0.05, *d* = 0.81) and UM conditions (*M*_diff_ = 1.43, *p* < 0.05, *d* = 0.89). The main effect of time was not significant (*F*(3, 39) = 0.88, *p* = 0.460, *ηp*^2^ = 0.06). A significant main effect of music condition was also found (*F*(2, 26) = 8.09, *p* < 0.01, *ηp*^2^ = 0.38), indicating that the FM condition elicited higher pleasant emotion than the Con and UM conditions overall.

Analysis of variance revealed a significant difference across conditions for ΔPleasant emotion (*F*(2, 26) = 6.24, *p* < 0.01, *ηp*^2^ = 0.32), and post hoc comparisons indicated that the FM condition was significantly higher than the Con (*M*_diff_ = 1.14, *p* < 0.05, *d* = 0.69) and UM conditions (*M*_diff_ = 1.43, *p* < 0.05, *d* = 0.70).

### 3.5. Arousal

**Figure 7:**
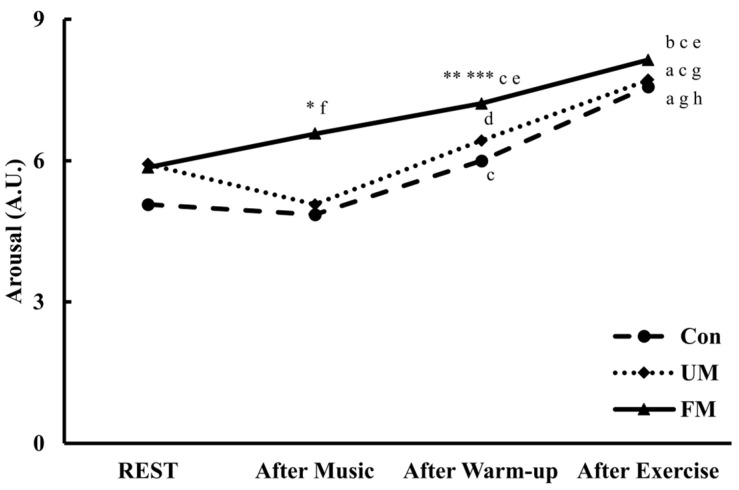
Changes in arousal (A.U.) across the 3 music conditions and time points. Values are presented as mean (*n* = 14). Two-way repeated-measures ANOVA revealed a significant condition × time interaction (*p* < 0.05). *: *p* < 0.05 vs. Con and UM; **: *p* < 0.01 vs. Con; ***: *p* < 0.05 vs. UM; a: *p* < 0.01 vs. After Warm-up; b: *p* < 0.05 vs. After Warm-up; c: *p* < 0.01 vs. After Music; d: *p* < 0.05 vs. After Music; e: *p* < 0.001 vs. Rest; f: *p* < 0.05 vs. Rest; g: *p* < 0.01 vs. Rest; h: *p* < 0.001 vs. After Music. Con: control; UM: unfamiliar language music; FM: familiar language music. Rest: at rest; After Music: after music listening; After Warm-up: after warm-up; After Exercise: after a 20 s maximal pedaling.

Figure 7 shows the temporal changes in arousal (A.U.) across the 3 music conditions and time points. Analysis of variance revealed a significant interaction across music condition and time (*F*(6, 78) = 2.65, *p* < 0.05, *ηp*^2^ = 0.17). The simple main effect of music condition was significant for After Music (*F*(2, 26) = 6.97, *p* < 0.01, *ηp*^2^ = 0.35) and After Warm-up (*F*(2, 26) = 8.98, *p* < 0.01, *ηp*^2^ = 0.41). Post hoc comparisons revealed that in the After Music condition, the FM condition showed significantly higher values than both the Con (*M*_diff_ = 1.71, *p* < 0.05, *d* = 0.88) and UM conditions (*M*_diff_ = 1.50, *p* < 0.05, *d* = 0.68). Furthermore, in the After Warm-up condition, the FM condition was significantly higher than the Con (*M*_diff_ = 1.21, *p* < 0.01, *d* = 1.08) and UM conditions (*M*_diff_ = 0.79, *p* < 0.05, *d* = 0.70). The simple main effect of time was significant for the Con (*F*(3, 39) = 16.76, *p* < 0.001, *ηp*^2^ = 0.56), FM (*F*(3, 39) = 18.32, *p* < 0.001, *ηp*^2^ = 0.58), and UM conditions (*F*(3, 39) = 11.97, *p* < 0.001, *ηp*^2^ = 0.48). Post hoc comparisons revealed that in the Con condition, After Exercise was significantly higher than Rest (*M*_diff_ = 2.50, *p* < 0.01, *d* = 1.17), After Music (*M*_diff_ = 2.71, *p* < 0.001, *d* = 1.71), and After Warm-up (*M*_diff_ = 1.57, *p* < 0.01, *d* = 1.17), and After Warm-up was significantly higher than After Music (*M*_diff_ = 1.14, *p* < 0.01, *d* = 1.20). In the FM condition, After Exercise was significantly higher than Rest (*M*_diff_ = 2.29, *p* < 0.001, *d* = 1.48), After Music (*M*_diff_ = 1.57, *p* < 0.01, *d* = 0.98), and After Warm-up (*M*_diff_ = 0.93, *p* < 0.05, *d* = 0.81), while After Warm-up was significantly higher than Rest (*M*_diff_ = 1.36, *p* < 0.001, *d* = 1.35) and After Music (*M*_diff_ = 0.64, *p* < 0.01, *d* = 0.86). Furthermore, After Music was significantly higher than Rest (*M*_diff_ = 0.71, *p* < 0.05, *d* = 0.78). In the UM condition, After Exercise was significantly higher than Rest (*M*_diff_ = 1.79, *p* < 0.01, *d* = 1.17), After Music (*M*_diff_ = 2.64, *p* < 0.01, *d* = 1.11), and After Warm-up (*M*_diff_ = 1.29, *p* < 0.01, *d* = 1.02), and After Warm-up was significantly higher than After Music (*M*_diff_ = 1.36, *p* < 0.05, *d* = 0.70).

Analysis of variance revealed a significant difference across conditions for ΔArousal (*F*(2, 26) = 4.17, *p* < 0.05, *ηp*^2^ = 0.24), but post hoc comparisons indicated no significant differences across conditions (FM vs. Con: *M*_diff_ = 0.93, *p* = 0.055, *d* = 0.62; FM vs. UM: *M*_diff_ = 1.57, *p* = 0.055, *d* = 0.71).

### 3.6. RPE

**Figure 8:**
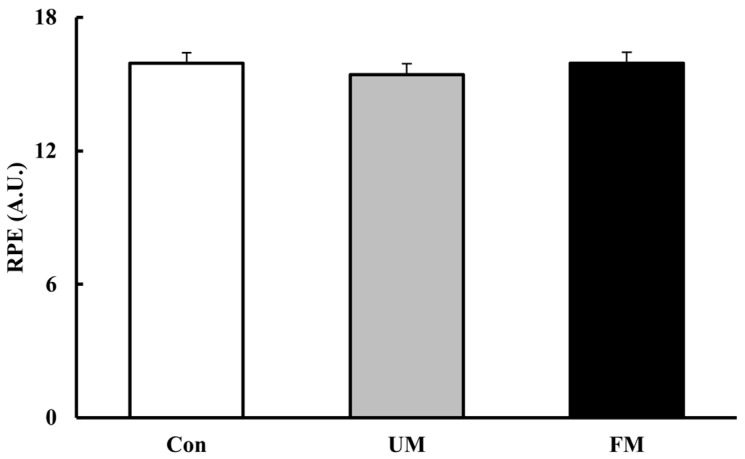
Rating of perceived exertion (RPE, A.U.) across the 3 music conditions after 20 s maximal pedaling. Values are presented as mean ± SEM (*n* = 14). A one-way repeated-measures ANOVA revealed no significant differences across conditions (*p* = 0.332). Con: control; UM: unfamiliar language music; FM: familiar language music. Bar colors indicate stimulation level: white (Con), gray (UM), and black (FM).

Figure 8 shows RPE (A.U.) across the 3 music conditions. Analysis of variance revealed no significant differences across conditions (*F*(2, 26) = 1.151, *p* = 0.332, *ηp*^2^ = 0.08).

### 3.7. Prefrontal Hemodynamics

Table 2 shows ΔHbO_2_ (mM·mm) and ΔHbR (mM·mm) across the 3 music conditions. Analysis of variance revealed no significant differences in ΔHbO_2_ across conditions (*F*(2, 26) = 0.54, *p* = 0.590, *ηp*^2^ = 0.04). Furthermore, analysis of variance revealed no significant differences in ΔHbR across conditions (*F*(2, 26) = 0.51, *p* = 0.609, *ηp*^2^ = 0.04).

### 3.8. Autonomic Nervous System Activity

Table 2 shows ΔLF/HF (A.U.) across the 3 music conditions. Analysis of variance revealed no significant differences in ΔLF/HF across conditions (*F*(2, 26) = 1.99, *p* = 0.157, *ηp*^2^ = 0.13).

### 3.9. Music and Lyrics Motivation Components

Table 3 shows BMRI-3 scores (A.U.) and scores for the motivation components of lyrics (A.U.). The paired *t*-test revealed that the BMRI-3 score was significantly higher in the FM condition compared to the UM condition (*t*(13) = 6.42, *p* < 0.001, *d* = 1.72, *M*_diff_ = 17.64), confirming that self-selected music with familiar language lyrics perceived as motivational indeed possessed objectively higher motivational characteristics. The paired *t*-test results for the motivation component scores of the lyrics showed that the FM condition was significantly higher than the UM condition (*t*(13) = 5.88, *p* < 0.001, *d* = 1.57, *M*_diff_ = 3.36). Table 4 shows the questionnaire survey responses.

## 4. Discussion

This study is the first to examine how lyric comprehension in a familiar language influences sprint performance and psychological responses when music tempo is controlled. Listening to self-selected familiar language music perceived as motivational significantly enhanced relative peak power during a 20 s maximal pedaling test. Motivation and arousal increased after music, and pleasant emotion remained consistently elevated throughout the session, whereas mean power showed no differences across conditions. These findings indicate that lyric comprehension, rather than tempo or rhythm alone, contributes to ergogenic and psychological responses, although the effects of other musical elements cannot be entirely ruled out.

Previous studies have shown that fast-tempo or motivational music enhances motivation and performance [55]. In this study, the music tempo was standardized (120–150 bpm) to minimize tempo-related influences. Under these controlled conditions, enhanced peak power was observed only when participants listened to familiar language music perceived as motivational, suggesting that lyric comprehension may play a critical role in ergogenic responses beyond musical tempo.

The BMRI-3 results showed that self-selected music with familiar language lyrics perceived as motivational received significantly higher motivational ratings than unfamiliar language music, indicating a clear psychological distinction across the two conditions. Ratings on the item “The lyrics of the music I listened to increased my motivation” were also significantly higher in the familiar language condition. In this condition, 13 of 14 participants reported understanding the lyrics, while one did not. However, even when this participant was excluded (*n* = 13), the significant difference in peak power across FM and Con conditions for peak power remained (*p* = 0.007), supporting the robustness of the main conclusion. In contrast, none of the participants understood the lyrics or correctly identified the language in the unfamiliar language condition. These findings suggest that understanding lyrics plays a more important role in enhancing motivation than the tempo of the music.

All participants rated their familiar language selections as “liked”, whereas most rated the unfamiliar language songs as “not liked”, suggesting a preference bias between conditions. However, the present study did not quantify music preference; therefore, it is plausible that familiarity and lyric comprehension jointly contributed to both liking and motivation. In the FM condition, all participants rated music as “music they liked”, while in the UM condition, 12 participants (excluding 2) rated it as “music they did not like”, indicating clear differences in preference levels across conditions. Because participants used self-selected music, the isolated effect of lyric comprehension cannot be completely excluded. Future research using standardized stimuli is needed to clarify the distinct contributions of lyrics, melody, and preference.

In the FM condition, motivation and arousal after music were higher than in the other conditions, and pleasant emotion remained consistently elevated throughout the session. These psychological responses were consistent with the significant improvement in peak power observed in the FM condition. Previous studies have shown that listening to self-selected music enhances exercise performance and motivation [5,30], that lyrics in music play an important role in influencing mood and emotion [24,25], and that they may also increase motivation and improve exercise performance [27].

A detailed analysis of psychological indicators revealed that motivation and arousal showed no significant differences across conditions at rest; however, after music, the FM condition exhibited higher values compared to the other conditions, and improvements relative to rest were observed only in the FM condition. In contrast, a significant main effect of music condition was found for pleasant emotion, with the FM condition generally showing higher values than the other conditions throughout the session. The magnitude of change in ΔMotivation (*ηp*^2^ = 0.44) and ΔPleasant emotion (*ηp*^2^ = 0.32) was large, indicating robust psychological effects. Although ΔArousal did not reach statistical significance, its moderate-to-large effect size (*ηp*^2^ = 0.24) suggests that the limited sample size may have prevented statistical detection. These findings suggest that self-selected music with familiar language lyrics perceived as motivational appears to positively modulate emotional and motivational states.

Regarding the relationship between pre-exercise arousal and performance, previous studies have reported mixed results. Kasai et al. (2024) suggested that playing video games increased arousal, leading to improved peak power during subsequent maximal pedaling exercise [56]. In contrast, Kasai et al. (2023) reported that listening to music before exercise that enhanced motivation improved peak power, whereas arousal levels did not differ among music conditions [55]. Collectively, these findings suggest that not only the magnitude of arousal, but also the quality of arousal-inducing stimuli and cognitive factors such as lyric comprehension may exert distinct effects on exercise performance.

Since no difference in mean power was observed across conditions, the effect of pre-exercise music listening may not influence sustained exercise capacity. Previous studies indicate that mean power during maximal pedaling exercise reflects sustained power output capacity [57], and that listening to music during exercise can sustain power output [28]. In this study, music was listened to only before exercise. This difference in music listening timing may have influenced sustained power output, potentially explaining why mean power was unaffected by whether music was listened to or the type of music listened to.

Regarding the effect of lyrics on exercise performance, Ballmann et al. (2025) examined how censoring explicit language in music influences resistance exercise performance and found that unaltered lyrical content enhanced repetitions during exercise [27]. In contrast, this study focused on lyric comprehension and had participants listen to music before exercise, yet no change in mean power was observed. These results suggest that while psychological changes induced by pre-exercise music listening may influence peak power output, their effect on sustained power output is limited. Continuous music listening during exercise may therefore be necessary to elicit endurance-related benefits.

No significant differences were observed in RPE across conditions. Terry et al. (2020) reported that music can reduce RPE during moderate-intensity exercise, primarily through attentional distraction from unpleasant sensations [2]. However, they also emphasized that this perceptual benefit tends to diminish as exercise intensity increases, particularly during high-intensity or maximal-effort tasks [2]. This theoretical framework aligns with the present findings: because the 20 s sprint test in this study required near-maximal effort, attention was likely directed almost entirely toward task-related sensations and physical strain, leaving little opportunity for the dissociative effects of music [2]. Consistent with our previous findings [55], the effect of pre-exercise music on RPE appears to be limited during short-duration, high-intensity sprinting.

To explore the physiological mechanisms underlying changes in sprint performance, this study measured prefrontal hemodynamics (ΔHbO_2_ and ΔHbR) and heart rate variability (LF/HF). Previous research has suggested the involvement of both the central nervous system and the autonomic nervous system (ANS) in music-induced performance enhancement. This study focused on prefrontal activity, which plays a central role in executive function and cognitive control [58], and on sympathetic modulation, whose activity increases during music listening [15]. However, no significant differences were observed across conditions in ΔHbO_2_, ΔHbR, or ΔLF/HF, indicating that the physiological responses measured in this study were not strongly modulated by music condition. In this exploratory study, psychological indicators (ΔMotivation: *ηp*^2^ = 0.44; ΔPleasant emotion: *ηp*^2^ = 0.32; ΔArousal: *ηp*^2^ = 0.24) showed large-to-moderate effect sizes, whereas physiological indicators (ΔHbO_2_, ΔHbR, and ΔLF/HF) showed small-to-moderate effects. These findings collectively suggest that the improvements in sprint performance observed in the FM condition were mainly mediated through psychological and neurocognitive mechanisms, rather than through overt physiological activation.

Although psychological indices such as motivation and pleasant emotion increased markedly under the FM condition, changes in arousal were relatively small, and no significant alterations in ΔLF/HF were detected. Previous studies have shown that increases in subjective arousal are generally accompanied by enhanced autonomic activation, particularly sympathetic dominance [59,60]. However, in the present study, it is possible that listening to familiar language motivational music contributed to emotional stabilization [61] and cognitive focus [59], such that the increase in subjective arousal did not reach the physiological threshold necessary to elicit measurable autonomic changes.

It is also conceivable that familiar language lyrics evoke an immediate sense of emotional resonance or excitement even before conscious comprehension occurs; however, such subtle psychological reactions may not have manifested as measurable physiological changes under the present experimental conditions. To capture such transient neural responses and to clarify the temporal and neurocognitive mechanisms underlying the effects of lyric comprehension, the combined use of fNIRS and electroencephalography (EEG), which offers superior temporal resolution, may be effective. Furthermore, measuring peripheral biomarkers such as plasma dopamine concentrations could allow for a more detailed assessment of mesolimbic reward system activation during music listening. Such a multimodal neurophysiological approach may enable more precise detection of neural responses related to lyric comprehension and affective processing, and facilitate linking these responses to neurochemical activity within the reward circuitry.

In contrast to the significant increases in maximal power, motivation, and pleasant emotion observed when listening to self-selected music with familiar language lyrics perceived as motivational, the absence of changes in physiological indices suggests that lyric comprehension may influence sprint performance primarily through specific psychological and neurocognitive mechanisms rather than broad physiological activation. Future studies incorporating multimodal physiological measures and larger sample sizes will be essential to further clarify the interaction between psychological and autonomic responses in lyric-based music effects.

This study has several methodological limitations that should be acknowledged.

First, because participants selected their own music, the musical stimuli differed across individuals in elements such as melody, rhythm, and instrumentation, which may have affected the results. Future studies should standardize these musical factors to better isolate the effect of lyric comprehension.

Second, the study sample consisted of relatively homogeneous participants—healthy young men with regular exercise habits—which limits the generalizability of the findings. Moreover, participants were recruited through convenience sampling from the researchers’ local university networks, which may have introduced selection bias and further limited the external validity of the results. Future studies with larger and more diverse samples are warranted to determine whether similar effects occur in females or other age groups.

Third, the lack of assessment of participants’ habitual use of music during exercise. Information on participants’ habitual use of music during exercise was not collected, which may represent an additional factor influencing psychophysiological responses. Previous studies have shown that habitual or context-specific music use during exercise can modulate psychophysiological responses to both music and physical effort [5,62]. Because the present study focused on pre-exercise music listening, the extent to which such habits could influence the outcomes remains uncertain. Future research should therefore consider this factor when comparing pre-exercise and during-exercise music listening conditions.

Fourth, while Hindi was used as an unfamiliar language based on the participants’ backgrounds, the effects of other languages or language families remain unknown.

Fifth, the exercise test was limited to a single 20 s maximal pedaling effort, and the effects under different exercise modalities or durations have not been verified.

Sixth, because short-separation channels could not be analyzed for all participants due to signal instability, some degree of scalp blood flow contamination in the prefrontal hemodynamic data cannot be fully excluded [40,42].

In addition to these methodological constraints, several interpretational limitations should also be considered.

Seventh, differences existed in the characteristics of the psychological scales used in this study. A VAS allowing 0.1-unit evaluations was used for motivation, whereas the Affect Grid with 1-point increments was used for pleasant emotion and arousal. Differences in scale properties may have influenced measurement sensitivity, particularly for arousal assessed by the Affect Grid. Future studies could employ higher-resolution and continuous measures of arousal, combined with multimodal approaches such as fNIRS, EEG, and peripheral biomarkers, to more precisely evaluate transient neural and neurochemical responses related to lyric comprehension and to clarify the temporal changes and interrelationships between psychological and physiological responses during music listening. However, the Affect Grid has been widely used in exercise psychology research [20,32], supporting its practical validity.

Considering these limitations, future research should clearly distinguish between lyric comprehension and musical elements to verify their respective effects.

Ideally, standardized or multilingual versions of the same music could be used to unify musical elements and comparisons between “familiar language versions” with “unfamiliar language versions” could reveal the pure effect of lyric comprehension. Alternatively, music with equivalent motivational characteristics (e.g., BMRI-3 scores) from familiar and unfamiliar languages may be compared. Furthermore, large-scale studies involving diverse participants may be warranted to confirm the generalizability of these findings. Future research may also explore the effects of different exercise modalities (e.g., prolonged or intermittent exercise) and incorporate multimodal physiological assessments, such as fNIRS, EEG, and peripheral biomarkers, to deepen understanding of neurophysiological mechanisms. In addition, advanced statistical techniques, such as mediation analysis, could help clarify the relationships among lyric content, psychological indicators, and exercise performance.

## 5. Conclusions

Listening to self-selected music with familiar language lyrics perceived as motivational enhanced peak power output during subsequent maximal pedaling. Lyric comprehension likely played a key role in this ergogenic effect by increasing motivation, pleasant emotion, and arousal before exercise.

These findings highlight the practical importance of listening to motivational music in a familiar language to optimize performance in short-duration, high-intensity exercise. Incorporating familiar language music before maximal efforts may serve as a simple and effective psychological strategy to enhance performance.

## Figures and Tables

**Table 1:** The characteristics of the participants.

Variable	Mean ± SD
Age (years)	19.7 ± 1.7
Height (cm)	171.2 ± 3.1
Weight (kg)	65.8 ± 7.1
BMI (kg/m^2^)	22.4 ± 2.1
Exercise frequency	≥3 sessions/week

Values are presented as mean ± SD (*n* = 14).

**Table 2:** Mean changes in prefrontal hemodynamics (ΔHbO_2_, ΔHbR; mM·mm) and ANS activity (ΔLF/HF; A.U.) across the 3 music conditions.

Variable	Condition	Mean ± SEM	*F*(2,26)	*p*-Value	*ηp* ^2^
ΔHbO_2_ (mM·mm)	Con	−0.97 ± 1.45	0.54	0.590	0.04
	FM	0.68 ± 0.88			
	UM	0.28 ± 0.70			
ΔHbR (mM·mm)	Con	0.37 ± 0.74	0.51	0.609	0.04
	FM	−0.41 ± 0.34			
	UM	0.03 ± 0.35			
ΔLF/HF (A.U.)	Con	0.66 ± 0.30	1.99	0.157	0.13
	FM	−0.71 ± 0.62			
	UM	0.15 ± 0.54			

Values are presented as mean ± SEM (*n* = 14). A one-way repeated-measures ANOVA revealed no significant differences across conditions for ΔHbO_2_ (mM·mm), ΔHbR (mM·mm), or ΔLF/HF (A.U.) (*p* > 0.05). Con: control; UM: unfamiliar language music; FM: familiar language music.

**Table 3:** Motivational characteristics of music (BMRI-3 and lyric motivation scores, A.U.) across the FM and UM conditions.

Variable	Condition	Mean ± SEM	Mean Difference (95% CI)	*p*-Value
BMRI-3 (A.U.)	FM	35.79 ± 1.13	[33.35, 38.23]	*p* < 0.001
	UM	18.14 ± 2.16	[13.37, 22.91]	
Motivation component of lyrics (A.U.)	FM	5.29 ± 0.44	[4.37, 6.21]	*p* < 0.001
Did the lyrics of the music you	UM	1.93 ± 0.29	[1.31, 2.55]	
listened to increase your motivation?				

Values are presented as mean ± SEM with 95% confidence intervals [lower, upper] (*n* = 14). Paired *t*-tests indicated significant differences across FM and UM conditions for both BMRI-3 and lyric-derived motivational component scores (*p* < 0.001). FM: familiar language music; UM: unfamiliar language music.

**Table 4:** Questionnaire responses regarding music preference, lyric comprehension, and language recognition across the FM and UM conditions.

Condition	Question	Yes (*n*, %)	No (*n*, %)
FM	Q1. Did you like the music you listened to?	14 (100%)	0 (0%)
	Q2. Did you understand the lyrics of the music?	13 (92.9%)	1 (7.1%)
UM	Q1. Did you like the music you listened to?	2 (14.3%)	12 (85.7%)
	Q2. Did you understand the lyrics of the music?	0 (0%)	14 (100%)
	Q3. Did you recognize what language the lyrics were in?	0 (0%) ^a^	14 (100%)

^a^ One participant mistakenly answered “Yes (English),” although the song was in Hindi. Values are presented as the number of participants and percentages [*n* (%)]. FM: familiar language music; UM: unfamiliar language music.

## Data Availability

All data supporting the findings of this study are available within the manuscript. Additional data can be provided by the corresponding author upon reasonable request.
